# A Sick Benefit Scheme Reduces Unmet Healthcare Needs: An Natural Experiment in Seoul

**DOI:** 10.1093/eurpub/ckac131.271

**Published:** 2022-10-25

**Authors:** S Yu, D Moon, M Sohn, J Kim, H Chung

**Affiliations:** 1 BK21FOUR R&E Center for Learning Health Systems, Korea University, Seoul, South Korea; 2 Health Policy and Management, Korea University, Seoul, South Korea; 3 Center for Labour and Health, People’s Health Institute, Seoul, South Korea; 4 Division of Health and Medical Sciences, The Cyber University of Korea, Seoul, South Korea

## Abstract

South Korea experiences four times more unmet healthcare needs than OECD countries (11.6% and 2.6% respectively). Unmet healthcare needs are caused by the double burden of direct and indirect costs including income loss, and OECD countries operate a sickness benefit scheme to resolve sudden loss of pay. Seoul introduced the first sickness benefit system, Seoul-Type Paid Sick Leave Support (hereinafter Seoul Sick Leave), for self-employed national healthcare insurance subscribers to reduce the rate of unmet healthcare needs. By comparing the amount of increasing medical expenses between the beneficiary and non-beneficiary before (2018) and after (2019-2020) the introduction of the system, the study was intended to confirm the reduced unmet healthcare needs. This study used data from the National Health Information Database (NHID) and the difference in differences (DID) analytic framework. 96 and 121 patients were included in benefit and non-benefit cohorts, respectively. As a result, the beneficiary group’s expenses were smaller than those of the non-beneficiary group (coef.=-1.24, p = 0.026). However, the beneficiary group had a greater amount of increase in hospitalization expenses before and after the introduction than the non-beneficiary group did (coef.=1.66, p = 0.005). Our finding showed that the Seoul Sick Leave helped the precarious workers as they were able to use inpatient services when they needed. If it is to be scaled up to the national level, it should be applied all people to enhance universal health insurance in Korea.

**Key messages:**

• Identified the effectiveness of the first sickness benefit system as it helped the precarious workers as they were able to use inpatient services when they needed.

• By financially supporting them, the Seoul Sick Leave support can achieve health promotion through early detection and treatment.

